# Effectiveness of Health Promotion Curriculum in Enhancing Non-communicable Diseases Health Literacy in Indian Youth

**DOI:** 10.7759/cureus.22530

**Published:** 2022-02-23

**Authors:** Ankita R Shah, Rahul M Jindal, Malavika A Subramanyam

**Affiliations:** 1 Social Epidemiology, Indian Institute of Technology Gandhinagar, Gandhinagar, IND; 2 Surgery, Uniformed Services University of the Health Sciences, Bethesda, USA

**Keywords:** health promotion, india, college youth, modifiable risk factors, theory-based health literacy, non-communicable diseases, health literacy

## Abstract

Background

There is a theory-praxis gap related to health literacy interventions focused on non-communicable diseases (NCDs) among young people. We designed an NCD curriculum and investigated its’ effect on health literacy in non-medical, non-nursing college students in India. We deliberately selected non-medical and non-nursing college students (age 17 to 22 years) as we hypothesized, they would have a minimum baseline knowledge of NCDs.

Methods

We initially carried out a pilot study on 85 students in a four-day-long workshop (32 teaching hours) using empirically developed health literacy instrument. We administered the curriculum to 120 randomly selected students in four colleges, while 50 students were assigned to the comparison group. The curriculum was given over four days for a total of 32 hours. Approval was sought to give four credits for completion of the course. Each lecture comprised didactics, followed by discussion, and skills testing of measuring blood pressure and blood sugar. Health literacy was measured using a specifically designed tool at baseline and endline. The difference in health literacy scores between the two time-points (timepoint 1: before delivering the curriculum, time-point 2: at the end of four days of training) was analyzed using the t-test. Multiple linear and Poisson regression models were used to account for covariates.

Results

The average difference between the intervention and the control group in baseline scores was 0.05% points (unpaired t-test statistics: -1.36, degrees of freedom 103.15, p>0.05). The same at endline was 20.59% points (unpaired t-test statistics: -11.31, degrees of freedom 138.14, p<0.001). The endline to baseline difference in health literacy scores was 18.54% points higher in the intervention group versus the control group (unpaired t-test statistics: -10.88, degrees of freedom 161.32, p<0.001). The difference-in-difference scores remained significant after accounting for college setting and baseline score (Multivariable linear regression model, β: 19.62% points, p<0.001). None of the socio-economic characteristics were significantly associated with the difference in the difference scores, independent of the intervention effect. The proportion of participants scoring 40% or above on the health literacy measure at endline was significantly higher in the intervention versus the control group (p<0.001).

Conclusions

We provide empirical data to support the incorporation of NCDs as a credit course in college curricula in low- and middle-income countries. Our findings showed that a theory-driven skills-focused curriculum may be a tool for enhancing NCD health literacy in Indian youth from diverse academic and socio-economic backgrounds.

## Introduction

Non-communicable diseases (NCDs) are a critical global concern due to significant mortality and morbidity [1]. NCDs generally have a non-infectious origin, tend to be of long duration and are the result of a combination of genetic, physiological, environmental and behavioral factors [1]. Four major NCDs are cardiovascular diseases, cancers, chronic respiratory diseases and diabetes [1]. The significant socio-economic impact associated with NCD threatens sustainable development, necessitating action at global, regional, and national levels [1]. India is experiencing a rapid epidemiological transition, with the rising burden of NCDs as well as major risk factors underlying NCDs [2]. NCD burden in India increased from 48% to 75% between the years 1990 and 2016 [2]. Currently, NCDs are among the top five causes of morbidity and mortality in India [2]. Specifically, the contribution of five major behavioral and metabolic risk factors; unhealthy diet, high blood pressure, high blood sugar, high cholesterol and obesity in the total disease burden increased dramatically from 10% in 1990 to 25% in 2016 [2]. This is complicated by disease onset a decade earlier in Indians compared to residents of high-income countries, increasing their risk of adverse medical and socio-economic consequences [2].

Youth, being at a transformative age, characterized by experimentation and susceptibility to risk behaviors, are considered to be at an increased risk of developing adverse health behaviors such as unhealthy dietary practices, tobacco use, harmful use of alcohol and sedentary habits [3]. Harmful effects of these behaviors lead to NCDs and associated poor health in later life [3]. Estimates suggest that approximately 70% of premature deaths in adulthood are the result of health-related behaviors that were initiated in childhood and adolescence [3]. It has been shown that the youth in India have low levels of awareness about NCD [4], and skills to make healthy lifestyle choices [5]. There is a high prevalence of NCD- related risk behaviors including dietary behaviors (such as consumption of fast food, green leafy vegetables, fruits), physical activity, and consumption of alcohol or tobacco [4], and metabolic risk factors in this age group [6,7]. It has also been shown that this age group is exposed to a barrage of media messages that influences their health behavior, which makes it imperative to empower youth to make informed lifestyle choices [8].

As a central goal of health education, health literacy is widely recommended as a cost-effective measure to reduce preventable NCD risk factors [9]. While health literacy is an evolving concept and a range of definitions are used, there is a general consensus that health literacy is a complex interconnected set of abilities, which goes beyond the ability to simply read pamphlets, make appointments or compliance with doctors' prescription [10]. Taking a comprehensive view, the World Health Organization has defined health literacy as “the personal, cognitive, and social skills which determine the ability of individuals to gain access to, understand, and use the information to promote and maintain good health [10].” Health literacy conceptualized in this way implies the achievement of a level of knowledge, skills and confidence to take action toward changing personal lifestyles as well as living conditions that improve personal and community health [10]. Taken together, health literacy enhances individuals’ ability to participate in decision-making processes in various aspects of life concerning individual and community health [10]. For instance, the ability to apply critical health literacy skills to critically reflect on targeted industry marketing of products such as tobacco or sugar-sweetened beverages or products claiming to be ‘healthy’ such as protein supplements, weight-loss products, low-fat packaged snacks, or ‘low-calorie’ foods, etc., and empowered to make healthier choices for themselves or family and individuals in the social network. At higher levels of health literacy, groups of people might be empowered to hold the governments accountable for making health-promoting policies [10].

Thus, situated within the paradigm of health promotion, health literacy contributes toward the promotion of equity by improving people’s access to health information. Health literacy skills empower young people to make informed health decisions throughout their life [10]. Moreover, health literacy in youth has the potential to spread health-related messages to their families, peers, and others in their social network, thus acting as agents of social change [11]. Actionable recommendations based on context-specific health literacy interventions are emphasized to prevent and control NCDs and related risk factors [12].

A way to develop health literacy skills in young people is to incorporate them into formal education [13]. Lifestyle interventions with school and college-going youth in India have shown the potential to increase awareness about NCD and related risk factors [11,14-17]. However, only a few of these studies have included a comparison group to evaluate their effectiveness. Most of these interventions have primarily targeted health behavior change, with health education as a primary component. Improved health literacy is conceptualized as a primary outcome of health education within the broader context of health promotion [10]. Research on health education with a health literacy focus is at an early stage in low- and middle-income countries. Although we found several studies assessing the extent of health literacy and its association with health status, especially in areas of dental and mental health, child undernutrition, and NCD self-management, we could not find any intervention study that operationalized health literacy in the Indian context.

Current efforts toward addressing health awareness, education, communication in general and specifically addressing NCD in the Indian context [11,14-17], include online resources, and school curricula which are, however, limited by one or more of the following:

1. Minimal emphasis on imparting skills for practical application of health information.

2. Limited ability to treat a topic in a contextually relevant, holistic manner.

3. Limited emphasis on critical thinking to help decision-making when presented with health information from various sources.

4. Over-simplistic and incomplete explanations of biological processes underpinning disease outcomes. 

5. Inadequate sensitization about structural determinants of health. 

6. No explicit mention of theoretical underpinnings guiding the curriculum design, and limited incorporation of health literacy in the purpose and methodology of health education and communication.

Furthermore, a review of recent policy documents on education- National Education Policy 2020 [18] and health- National Health policy 2017 [19] suggested that there is little emphasis on integrating comprehensive skills-focused health education as an essential and core part of formal education in India pointing to lack of policy focus. Limited policy and financial emphasis might partly explain the lack of emphasis on the development and implementation of skills-focused curricula in schools and colleges. 

Our literature search confirmed that nutrition education curricula taught in secondary schools in India have been critiqued by teachers, parents and participants as being outdated, inadequate in imparting practical skills and emphasizing rote learning [20]. This highlights the theory-praxis gap related to health literacy interventions across age groups, especially in NCD-related literacy interventions. We, therefore, investigated the effect of a contextually-relevant, theory-informed, health literacy curriculum on NCD literacy among non-medical and non-nursing college students in the State of Gujarat, India. We planned a health promotion intervention study with an intervention group and a matching comparison group to evaluate the effectiveness of the curriculum. We deliberately selected non-medical and non-nursing college students as we hypothesized they would have minimal knowledge of NCDs. 

This article was placed on the pre-print server - Research Square, 10.21203/rs.3.rs-1170895/v2 on December 15, 2021. 

## Materials and methods

We have used the CONSORT guidelines to report our trial [21].

Specific objectives

The main objectives are to design, deliver, and test the effectiveness of a health literacy curriculum in increasing NCD-related health literacy as measured by a specifically designed questionnaire in college students in Gujarat, India. We planned a quasi-experimental health promotion intervention with an intervention group and a matching comparison group to evaluate the effectiveness of the curriculum.

Theoretical premise

**Figure 1:**
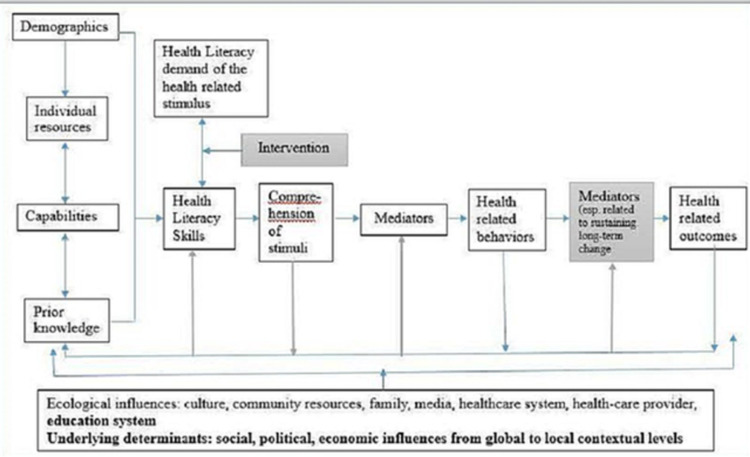
Health literacy skills framework Adapted and modified from Squiers et al. [22]. All the arrows and boxes in grey and text in bold represent our modifications.

The theoretical premise of the curriculum was health literacy within the health promotion paradigm, viewed through a social epidemiological lens. We considered health literacy as a key determinant of health and health equity (10). We adopted the Health Literacy Skills (HLS) Framework proposed by Squiers et al. [22] with several conceptual modifications (Figure 1) based on the eco-social theory [23] and Nutbeam’s [10] tripartite model of health literacy. 

We have modified the HLS framework [22] in the following domains:

1. The framework posits that ecological influences moderate components of conceptual framework in several ways. We extended this proposition to an upstream level, emphasizing the influence of broader social, political, and economic influences, from global to contextual level in producing and maintaining systematic health differences within and across populations through a range of mechanisms and pathways.

2. We contend that engaging in a health behavior does not necessarily lead to improved health outcomes for two reasons. First, health behavior needs to be sustained before health impact becomes visible at the community level; however, the determinants of sustenance of behavior appear to be different from the determinants of initiation of behavior change and may remain unaddressed. Second, a range of health determinants other than behavior change such as the health system, environment, food policies and other upstream determinants can affect health outcomes, which may remain unaltered.

3. We incorporate critical health literacy as a dimension of health literacy skills. As Nutbeam posits, adequately emphasizing critical health literacy has the potential to act laterally to promote social action that impacts social determinants of health.

4. We extend the concept of the dynamic nature of health literacy in the HLS framework and propose that health literacy skills further amplify this through a feedback loop from a comprehension of stimuli to knowledge.

Content selection

Curriculum content was informed by the theoretical framework. We primarily focused upon two health conditions i.e. high blood pressure and diabetes mellitus, both have a high prevalence in India [2,24]; and preventable risk factors, dietary modifications and physical activity.

Development of the curriculum

We consulted several sources such as textbooks and peer-reviewed literature in clinical medicine, human physiology, health promotion, disease prevention, behavioral risk factors, food and nutrition science, exercise physiology, health behavior change theories, and health education. We reviewed regional variations in food practices across India, as well as social causes of health disparities. We referred to food labeling laws, including their historical context in Indian and global contexts [25]. The overview of curriculum modules is described in Table 1.

**Table 1:** Overview of curriculum modules

No.	Title	Contents
1	NCD: concept and concerns	Difference between communicable and non-communicable diseases, general characteristics of NCDs, current rates of prevalence of different NCDs globally and in India (disability and deaths), social and economic cost involved with NCDs, role of prevention in reducing this burden.
2	Risk factors: concept and importance	What do we mean by risk factors, how are these associated with NCDs, different types of risk factors, role of risk factors in NCDs prevention
3	DM: what, how and why	Role of sugar in blood, role of insulin on blood sugar level and in the body, what occurs to blood sugar in DM, what occurs to insulin in DM, different risk factors involved in occurrence of insulin resistance, important complications of poorly controlled DM, available treatment options, role of controlling modifiable risk factors in reducing risk of getting DM and related complications, and in improving treatment efficacy, dispelling common myths associated with DM
4	DM: measuring capillary blood sugar	Overview of laboratory tests to detect DM, and related complications, role of blood sugar monitoring in effective control of DM, learning to measure capillary blood sugar (and interpret obtained values) using digital blood glucose measuring device
5	HT: what, how and why	What is blood pressure, role of blood pressure in the body, overview of factors involved in creation of blood pressure, physiological fluctuations in blood pressure levels (during exercise, in acute stress), what is HT, factors involved in its occurrence, important complications of poorly controlled HT, available treatment options, role of controlling modifiable risk factors in reducing risk of developing HT and related complications, and in improving treatment efficacy, dispelling common myths associated with HT
6	HT: Measuring blood pressure using digital machine	Method of detecting HT, overview of tests to detect HT related complications, role of blood pressure monitoring in its effective control, learning to measure blood pressure (and interpret obtained values) using digital blood pressure measurement device,
7	Atherosclerosis and Dyslipidemia: what, how and why	What is atherosclerosis, how does it develop, its involvement with HT, role of cholesterol in blood and in body, normal blood cholesterol levels, what is dyslipidemia, how does it occur, its association with atherosclerosis, obesity and insulin resistance, dispelling common myths associated with dyslipidemia
8	Overweight and obesity: what, how and why	What is it, important risk factors associated with its occurrence, from where does the excess fat come and how does it get deposited in the body, role of caloric management in its prevention and control, health implications of having excess weight, health benefits of maintaining optimal weight, dispelling common myths associated with its prevention, control and causation
9	Obesity: anthropometry	Measuring body weight, height and waist circumference (WC), calculating body mass index (BMI) and learning to interpret the obtained values of BMI and WC
10	Diet and dietary modifications	Concept of macro and micronutrients in food. Macronutrients: role in the body, types and sub-types and specific characteristics of each, concept of glycaemic index and glycaemic load of food items, role of sugar and different subtypes of fat on blood cholesterol level and in occurrence of atherosclerosis, dietary sources of each macronutrient, relative advantages and limitations of each source, identifying food items that are good sources of each macronutrient available to and consumed by the participants in different contexts, critical analysis of relative advantages and limitation of each food item, identifying healthier food items and alternatives for not-so-healthy food items within different contexts, dispelling common myths associated with diet and food.
11	Making sense of food labels	Food labeling guidelines in India, meanings of commonly used abbreviations, codes and alternative names indicating similar ingredients, interpreting, ingredients list and nutrition facts on the food label, identifying ingredients with not-so-healthy nutrients on the food label, critically evaluating credibility of nutrition/health claim made for the food item, selecting healthier food item from given option, based on information available on the food label.
12	Physical activity and exercise	health implications of leading a physically inactive life, how do we get health benefits from engaging in physical activity (especially related to health conditions covered here), different types of physical activities (including overview of different types of exercise, and specific benefits from each type) with examples of routine activities and household chores, recommended minimum levels of physical activity for all age-groups, ways to incorporate physical activity in daily routine, precautions while engaging in physical activities, dispelling common myths associated with physical activity and exercise
13	Critical perspectives NCDs and related risk factors	Creating sensitization towards role of various social, economic, political and legal factors at different levels (from global to local); including powerful forces such as market, urbanization and globalization; in creation, sustenance and reinforcement of the unequal distribution of NCDs related risk factors, disease burden and resources including awareness and skills to reduce exposure to the same across population by discussing examples related to food items and nutrition products’ marketing, food labeling laws, health care access and affordability, lack of policies related to health education, and so on.

Content appropriateness of the curriculum

The content was developed and peer-reviewed by a team of experts, which included social epidemiologists and public health practitioners with a background in medicine, health education, nutrition, and health promotion. The team reviewed the comprehensiveness and relevance of the contents included as a part of the curriculum in each module. They also assessed the alignment of the contents included in the curriculum with the learning objectives defined for each module. 

Developing the evaluation tool

A number of approaches and instruments have emerged for measuring health literacy [26]. However, none were sensitive and specific to objectively assess the impact of our intervention. We, therefore, developed an entirely new instrument to assess functional, communicative, and critical dimensions of health literacy. The final measure comprised 22 questions and participants were given 50 minutes to complete the test (Appendix). Cronbach’s alpha value for the items included in the measure was 0.74 indicating that the items included have internal consistency within an acceptable level.

Ethical considerations

The Institutional Ethics Committee of the host institute of the first author approved the study. Participants were given a consent form with information about the nature of the study, their role, the voluntary nature of their participation, and their right to withdraw from any session or the entire study without giving any reason. The first author verbally explained the contents of the consent form to the participants who were given an opportunity to seek clarifications before signing the form. All the study participants signed the written informed consent form before participating in the study. The study was registered on the ISRCTN registry (study ID ISRCTN28814900; date: August 28, 2020). Although our study is an intervention study, it did not involve outcomes related to behavior modification or therapeutic prevention or treatment. 

Pilot test

We carried out a pilot study in 85 students in a four-day-long workshop (total of 32 teaching hours) and carried out baseline and endline data collection using the empirically developed health literacy instrument. The curriculum we developed, required 16 to 18 hours for delivering the concepts and around 8 to 10 hours were required for carrying out in-class activities, discussion, and practical hands-on training under the supervision of the instructor. We required four hours to carry out the assessment at the baseline and endline. The four days over which we would get the necessary contact hours with the students were identified by the college administration based on their logistical feasibility. 

Sample size estimation

We defined our outcome of interest as the average difference in percentage scores between endline and baseline in the intervention and comparison groups. We used the formula to determine the sample size as suggested by Smith et al. [27]. The sample size required in each group to detect a specified difference D = μ1-μ2, with power specified by z2 and the significance level specified by z1 is given by 

n = [(z1+z2)2 (σ12 + σ22)] / (μ1-μ2)2

where σi (i=1,2) is the standard deviation of the outcome variable in both groups.

For 90% power, a significance level of 95% and the estimated value of SD of the outcome variable in both the groups at 15, the estimated sample size to detect a difference of 10% between both the groups was calculated to be 47 in each group. 

Sampling design

Undergraduate college students from the selected study sites who consented to participate in the study were included. After giving a brief introduction about the project to a group of students, the college administration gave us a list of students who had shown initial interest in participation. We randomly assigned two-thirds of the students (n=147) to the intervention group, while the rest of the students were automatically assigned to the comparison group (n= 63). Anticipating a higher loss to follow-up in the intervention group, we assigned a greater number of students to the intervention group as compared to the comparison group. The proportion of students who were lost-to-follow-up was, however, comparable across both the groups at approximately 18%. The age of participants ranged between 17 and 22 years.

Implementation of the intervention

The modular design of the curriculum was given over four days for a total of 32 hours from January 2018 to April 2018. Each lecture comprised the didactic section followed by discussion and skills testing of measuring BP and blood sugar. For the purpose of training, we used pre-calibrated electronic devices. Additionally, we emphasized that the purpose of measurements was to aid in the screening of a suspected case and home-based monitoring of the known case. They were given instructions regarding the next steps if a value did not fall within the normal range. The first author was accompanied by a research fellow who helped with logistical arrangements and recording observations and feedback. Hands-on training activities designed for each module were group-based activities involving demonstration of understanding the concepts, application of the learned concepts, identifying scenarios where learned concepts can be applied in day-to-day life for themselves and in the family or social network.

Data collection

Baseline and endline data in the intervention group were collected after delivering the modules. In the comparison group, we also collected baseline and endline data but no teaching was given.

Data analysis

Statistical analysis was carried out using STATA, version 12·1 [28]. We calculated the baseline and endline scores using the answer key we had developed along with the evaluation tool. The total score obtained was converted into a percentage using the maximum possible score as the denominator. Baseline and endline scores in percentage were treated as continuous variables. We calculated the difference in percentage points between endline and baseline scores by subtracting the baseline percentage from the endline percentage for all study participants. This difference-in-difference score was one of the two main outcome variables in this study. We carried out an unpaired t-test with unequal variances for comparing mean difference-in-difference scores between the two study groups.

We also carried out an intention-to-treat analysis using imputed values for the lost-to-follow-up in two ways:

1. Imputing a value of zero for the intervention group (n=27) and 8.99 (average difference value in the comparison group) for the comparison group (n=13).

2. Imputing a value of 8.99 (average difference value in the comparison group) for both the groups (n=40).

The average difference in health literacy scores (in percentage points) between the two study groups was compared using an unpaired t-test with unequal variances. We compared the background characteristics and baseline health literacy scores of the students in the analytical and lost-to-follow-up samples using the chi-square test and t-test, respectively.

Multiple linear regression models were fitted accounting for the background characteristics, study site, and baseline percentage as covariates. We retained study site and baseline percentage as covariates in our final model, as these were significantly associated with the outcome variable after adjusting for the main predictor variable.

We also used binary outcome indicators created from the continuous baseline and endline percentage scores by setting a 40% score as the cut-off value. Participants scoring 40% or above were grouped together. The binary outcome indicator measures the number of participants who scored 40% or above in both study groups, separately for baseline and for endline. Fixing 40% as the cut-off value was inspired by the percentage score cut-off score used by the colleges to declare a student as having passed a course - 35% of the total score in the final examination. We initially planned to set 50% as the cut-off, but no participants scored above 50% at the baseline; so, we fixed the cut-off value at 40% - higher than the passing cut-off value for participants taught in all the study sites. We used chi-square tests to compare the proportion of participants scoring 40% or above between the two study groups at each of the time points. We fitted multivariable Poisson regression models yielding Incidence Risk Ratios for the binary outcome to account for the covariates. We retained the study site and baseline percentage as covariates in our final model.

## Results

Intervention and comparison groups were comparable at baseline as shown in Table 2. The mean age of participants in the intervention group was 19.2 years (SD 1.8 years) and 19.6 years (SD 1.4 years) in the comparison group. 

**Table 2:** Frequency (%) distribution of background characteristics in intervention and comparison groups at baseline ^a^The total of all categories does not add up to the total in the group because of missing data for a few variables. ^b^Other Backward Classes, Scheduled Caste and Scheduled Tribes are official terms used in the Constitution of India to denote caste groups that are historically socially and/or economically marginalized.

Sr. no.	Characteristics	Intervention group n (column %)	Comparison group n (column %)	p value for chi-square test
1	Gender			
	Male	56 (46.67)	32 (64)	
	Female	64 (53.33)	18 (36)	0.04
2	Monthly parental income in Indian Rupees			
	20,000 or less	40 (37.74)	22 (57.89)	
	21,000-50,000	43 (40.57)	13 (34.21)	
	Above 50,000	23 (21.70)	3 (7.89)	0.05
3	Father’s education			
	Less than fourth standard	16 (14.29)	6 (13.64)	
	Fifth to 10th standard	30 (26.79)	13 (29.55)	
	Higher secondary (11^th^-12^th^) or some college	32 (28.57)	8 (18.18)	
	College and above	34 (30.36)	17 (38.64)	0.55
4	Mother’s education			
	Less than fourth standard	31 (27.68)	14 (31.82)	
	Fifth to 10th standard	43 (38.39)	15 (34.09)	
	Higher secondary (11^th^-12^th^) or some college	16 (14.29)	8 (18.18)	
	College and above	22 (19.64)	7 (15.91)	0.83
5	Caste background			
	General (most privileged)	56 (47.46)	22 (44.9)	
	Other Backward Classes^b^	43 (36.44)	18 (36.73)	
	Scheduled Castes and Scheduled Tribes (most marginalized)^b^	19 (16.10)	09 (18.37)	0.93
6	Academic performance in the previous academic year			
	60% and below	28 (26.17)	29 (28.43)	
	61%-70%	22 (20.56)	30 (29.41)	
	70% and above	57 (53.27)	43 (42.16)	0.21
7	Fitness related article/program on any kind of media in the past one month			
	Unexposed	53 (49.53)	9 (24.32)	
	Exposed	54 (50.97)	28 (75.68)	0.01
8	Diet related articles/program on any kind of media in past one month			
	Unexposed	57 (52.29)	13 (37.14)	
	Exposed	52 (47.71)	22 (62.86)	0.12
9	Fitness related activity in the next one month			
	Intended to engage	31 (29.81)	13 (37.14)	
	Unintended to engage	73 (70.19)	22 (62.86)	0.42
10	Diet-related activity in the next one month			
	Intended to engage	25 (24.27)	5 (14.29)	
	Unintended to engage	78 (75.73)	30 (85.71)	0.22
11	Fitness-related activity in the past one month			
	Did not engage	46 (42.20)	18 (47.37)	
	Engaged	63 (57.80)	20 (52.63)	0.58
12	Diet-related activity in the past one month			
	Did not engage	73 (67.59)	20 (57.14)	
	Engaged	35 (32.41)	15 (42.86)	0.26
13	Current place of residence			
	Rural	15 (13.27)	15 (34.09)	
	Urban	98 (86.73)	35 (65.91)	0.003
	Total	120^a^	50^a^	

**Table 3:** Average difference-in-difference (endline-baseline) score from a multiple regression model accounting for baseline score (n=170) **p<0.01, ***p<0.001

Covariate	Coefficient (95% confidence intervals)
Intercept	21.51 (15.76, 27.26) ***
Exposure to intervention	19.62 (15.59, 23.64) ***
Baseline score in percentage	-0.52 (-0.72, -0.33) ***

The average difference between the intervention and comparison group in the baseline health literacy scores was 0.05% points (p>0.05). The same at the endline was 20.59% points (p<0.001). The endline to baseline difference in health literacy scores between the study groups was 18.54% points higher in the intervention as compared to the comparison group (unpaired t-test statistics: -10.88, degrees of freedom 161, p<0.001).

Intention-to-treat analysis: Intention-to-treat analysis showed that the endline-baseline difference in health literacy scores were significantly different (p<0.001) between the two study groups; regardless of the value used to impute the difference score (Table 3). Furthermore, comparing the analytical and lost-to-follow-up samples suggested that students across both the groups were comparable on background characteristics (chi-square test, p>0.05) as well as on average health literacy scores in percentages at the baseline (t-test, p>0.05) (data not shown).

**Table 4:** Average (SD) difference in health literacy scores (in percentage points) in the two study groups found using intention-to-treat analysis The difference-in-difference scores remained significant after accounting for the baseline score (Multivariable linear regression model, β: 19.62% points, p<0.001).  None of the socio-economic characteristics were significantly associated with the difference in the difference scores, independent of the intervention effect.

Score: Difference between endline and baseline in percentage (All the participants included at the baseline are included in the analysis)	Intervention group mean (SD) (n= 147)	Comparison group Mean (SD) (n=63)	t value	Degrees of freedom	p-value for t-test	
						Difference score was imputed as zero in the intervention group and 8.99 in the comparison group for all the participants lost to follow-up or who did not have endline data in the comparison group.	22.47 (17.01)	8.97 (6.64)	-8.26	206.7	<0.001	
Difference score was imputed as 8.99 for all the participants lost to follow-up or who did not have endline data in the comparison group.	24.12 (15.06)	8.99 (6.64)	-10.11	207.8	<0.001	

The proportion of participants scoring 40% or above on the health literacy measure in both groups was comparable at baseline (Table 5). However, the proportion of participants scoring 40% or above was higher in the intervention group versus the comparison group at endline (p<0.001).

**Table 5:** Frequency (%) of study participants scoring 40% or above in the two study groups

Timepoint	Intervention group, n/N (%)	Comparison group, n/N (%)	P-value for Chi-square test
Baseline	6/120 (5.00)	4/50 (8.00)	>0.05
Endline	97/120 (80.83)	16/50 (32.00)	<0.001

Based on Poisson models, the incidence risk ratio of participants scoring 40% or above on the health literacy measure at endline was 2.44 times (p<0.05) higher in the intervention group versus the comparison group, after adjusting for baseline health literacy score in percentage points (Table 6).

**Table 6:** Incidence risk ratio (95% confidence intervals) of scoring 40% or above from a Poisson regression model accounting for baseline score (n=170). *p<0.05, **p<0.01, ***p<0.001

Predictor	Incidence risk ratio (95% confidence intervals)
Exposure to intervention (Comparison group as reference category)	2.44 (1.67, 3.55) ***
Baseline score (percentage)	1.02 (1.00, 1.03) *
Constant	0.43 (0.26, 0.70) **

## Discussion

Our findings suggested that our theory-based, context-specific, NCD-related curriculum significantly improved literacy on multiple dimensions among college-going youth in the state of Gujarat, India. The curriculum was effective for students with diverse academic and socio-economic backgrounds. To our knowledge, this is the first study in the Indian context to design an intervention using the health literacy framework and evaluate it using a comparison group. We, therefore, compared our findings with health education intervention studies that aimed at improving NCD awareness among youth. Gavaravarapu et al. [14]. carried out an intervention using a communication module to promote food label reading skills of school-going adolescents in Hyderabad, India. They reported 16.5% increase in food label reading skills in the intervention group (n =116), versus 1.85% increase in the comparison group (n = 59) (p<0.001). Strengthening food label interpretation skills was one of the learning objectives of our intervention. Although we did not separately assess this component, our overall finding of an average increase of 27.52% (SD: 14.65) in the intervention group as compared to an average 8.99% (SD: 4.83) increase in the comparison group, is consistent with the findings by Gavarvarapu et al. [14]. 

Classroom-based health and nutrition education intervention among college-going volunteers (n = 351) in Andhra Pradesh, India, reported a significant improvement of 11.36% in the average score of knowledge on nutrition and health in student volunteers after the intervention [11]. Significant improvement in the percentage of student volunteers answering correctly was observed in most items related to knowledge about nutrition and lifestyle diseases [11]. Chaudhary et al. [17] reported an increase in the percentage of school-going adolescents who demonstrated knowledge about major risk factors of NCD ranging between 24% and 47% after a single education session of 45 minutes. Our findings showed a 75% increase in the proportion of students who scored 40% or above in the intervention group as compared to 22% in the comparison group (p<0.001).

A study by Shah et al. [15] reported significant improvement ranging from 7% to 19% increase in the percentage of 15-18 years old school students who answered key nutrition, physical activity and NCD-related questions correctly after the intervention (n = 448) vs. before intervention (n = 539). Singhal et al. [16] reported a significant improvement in knowledge scores among 11th-grade school students after a multicomponent intervention on nutrition and lifestyle education for behavior modification. A randomly selected intervention group (n = 99) showed improvement in knowledge-related items as compared to the control group (n = 102).

We acknowledge the differences between these studies and our work, however, our findings are generally comparable with these studies [11,14-17]⁠. We believe that an engaging delivery of content, in-depth discussion, and hands-on activities helped sustain participants’ interest. Using analogies to explain complex bodily processes simplified learning, while contextualized examples and audio-visual material made the curriculum relevant. Taken together, this enhanced learning, which was reflected in increased health literacy was measured using a test of the application of learned material than a memory test.

Given that India presently has one of the world's largest youth populations exposed to a substantial risk of NCD, such prevention efforts have great potential to address this challenge. The intrinsic value of health education aimed at improving health literacy needs attention beyond its instrumental importance in bringing about behavior change. One limitation of a health education approach oriented toward behavior change is the intrinsic assumption that most of those who receive health education require behavior change in the immediate aftermath of such an intervention; and/or that health education needs to be targeted only toward those in need of behavior change. While a specific focus may result in improved behavioral outcomes, at least in the short term, it is essentially a high-risk approach to disease prevention. We argue that a population-level approach [29] aimed at improving health literacy in the entire population is a timely and worthwhile public health goal [10].

Health literacy, conceptualized as an essential life skill, has the potential to enhance an individual’s ability to lead a healthier life, by enabling him/her to make informed health choices. Along this line, age-appropriate content of health literacy might be integrated into education right from early childhood. For instance, curricula aimed at teaching health literacy skills to students and their care providers have been developed by the Centers for Diseases Control and Prevention [30]. The curricula are divided by grade levels from early childhood to university levels which can help childcare providers and educators in recognizing and responding to students’ their family members’ health information and communication needs [30]. While we tested the effectiveness of our curriculum among non-medical and non-nursing undergraduate college students, we hypothesize that it can be delivered to high school students (10th to 12th graders), young adults up to age 35 having at least 10th-grade education, as well as community-based frontline health workers.

Our study has several limitations. We measured endline scores immediately after completing our intervention. However, we did not measure the long-term impact of the intervention on health literacy. Higher scores may be due to the content remaining fresh in students’ memory. Importantly, it is worth recollecting that our health literacy measure was designed to test the students’ understanding and/or application of the concepts learned, rather than testing their memory of the concepts. We, therefore, believe that inflation in the health literacy scores due to this issue would be minimal. Conversely, we suspect that as the endline measurement was collected immediately post-intervention, the students did not have adequate time to reflect on the concepts they learned and how these might be applicable in their own lives. A measurement at a later time point could test whether students retained the concepts over a longer term and could apply some of these concepts in their everyday life. Future studies should consider a long-term follow-up of the study population with repeated measurements of health literacy scores post-intervention, supplemented by a qualitative inquiry into the participants’ views about their participation in the intervention. Another weakness is the empirical use of health literacy tools, which has not been validated. We hope our work will stimulate other researchers to undertake this task.

Our findings have shown that it is possible to improve NCD health literacy in Indian youth using this approach, independent of the participants’ socio-economic background and past academic performance. This intervention was also effective in improving health literacy scores across diverse academic backgrounds. However, our results pertain to a small geographic unit in India and need to be corroborated by studies with larger sample sizes. Nonetheless, our study demonstrated the potential for such an intervention and provides a tailor-made curriculum ready to scale up.

This is among very few studies globally, and to the best of our knowledge, the first study in the Indian context that addressed NCD-related health literacy comprehensively in healthy populations in a college setting, while establishing its effectiveness using a comparison group. Thus, we provide strong evidence of the feasibility and effectiveness of this health promotion approach in developing countries, where such efforts are urgently required to reduce the NCD burden. The major strength of this NCD-related health literacy curriculum is the integration of theories of social epidemiology and concepts of health literacy in the paradigm of health promotion within its’ theoretical foundation. This integration allowed us to modify the HLS framework, based on a holistic conceptualization of determinants of health literacy and its’ impact on health.

Our study has contributed to the existing body of literature by providing empirical support to the modified HLS framework in the developing country context. In essence, our intervention integrates sound theoretical premise with praxis by emphasizing skills-building in youth, of use in daily life. Notably, one of the characteristics of a successful health education curriculum is that they are theory-driven. Our curriculum covered the multidimensional concept of health literacy by including interactive and critical health literacy dimensions. Our curriculum is also unique in its content and approach to disease prevention and health promotion, which was rooted in the study’s theoretical premise. Our intervention was tailored for college-going youth and was sensitive to the local context in content design and skills-building. We emphasized practical skills and critical thinking through an interactive learning approach. As Nutbeam [10] argued, the difference in content and method of health education leads to differential learning outcomes.

We have objectively assessed health literacy multi-dimensionally using content and context-specific health literacy measure, thus addressing one of the limitations of research in this area [13]. This allowed us to measure health literacy specific to certain health problems such as NCD, and specific to different contexts, such as applying health knowledge while grocery shopping or interpreting health-related information when exposed to brochures, which are critical in health-related decision-making [13].

## Conclusions

Although the need for awareness on NCD with a health literacy focus is identified as an important component in NCD prevention and control, it remains a neglected area in the national dialogue. Scholars have identified the need to strengthen health education by including lifestyle awareness components targeting adolescents/youth in policies and programs related to NCD and adolescent health, including the need for adopting the health-promoting schools' framework endorsed by World Health Organization in India.

We strongly emphasize the need to prioritize comprehensive health literacy interventions, with a special focus on NCD in schools, colleges, including youth outside the formal education system. Our findings can serve as a basis for the incorporation of health literacy modules in college curricula giving appropriate credits for both theoretical knowledge and skills testing. The findings of our study are potentially a step toward policy change that supports health literacy in NCD in India and other low- and middle-income countries.

In the future, we aspire to scale up this intervention in several other colleges across India, using a randomized controlled design to establish robust evidence of the effectiveness of our health promotion intervention in improving NCD health literacy among college youth. This might help us argue for a policy-level change aimed at integrating health promotion as a part of training in educational settings.

## Appendices

Name of student:                                                                                                               

Date:

Please answer the questions given below. Read the instructions given with each question properly. Try to answer the descriptive questions in one or two lines. Please write your answers clearly and in bullet points.

1.     If you need to increase your protein intake, without significantly increasing your fat intake, which food will you prefer among these options?

a.     Whole milk                                                           c.  Dal-chapati

b.     Mutton curry                                                        d.  Egg omelette

2.     Choose healthier option from the given two food items. You plan to have either 6 slices of biscuits (approx. 50 gm) or 1 piece of besan laddu (approx. 50 gm). Use the nutritional information given for each food item. Clearly mention which food item would you choose and give at least one appropriate reason for choosing that particular food item.

a. Besan laddu:                                                                       b. Biscuits

a. Besan laddu

Nutrition information per 100g product

Carbohydrates               66 g

            Sugars              42 g

Protein 10 g

Fat       22 g

Saturated fatty acids                   10 g

Mono unsaturated fatty acids     11 g

Poly unsaturated fatty acids       1 g

Trans fatty acids                       0 g

Cholesterol                               16 mg

Energy                                    502 kcal

b. Biscuits:

Nutrition information per 100g product

Carbohydrates               65 g

            Sugars              22 g

Protein                         7g

Fat                               24g

Saturated fatty acids                 11 g

Mono unsaturated fatty acids    10.2 g

Poly unsaturated fatty acids      2.7 g

Trans fatty acids                       0 g

Cholesterol                               2mg

Energy                                     504 kcal

Your chosen food option: ………………………………………………………………………………………

Reason for choosing the same: ………………………………………………………………………………………

3.     Engaging in physical activities in a continuous spell of at least …………….. at a time gives health related benefits.

a.      10 minutes                                                      c. 15 minutes

b.     30 minutes                                                      d. 45 minutes

4.   List at least one health concern (lack or excess of any nutrient) associated with each food item given below.

Chicken Kabab: ………………………………………………………………………………………………

Packed fruit juices: …………………………………………………………………………………………….

Namkeen chivda (commercially packed): …………………………………………………………………

Bakery items such as pastry/cake: …………………………………………………………………

5.     A nutrition related claim mentioned on the food label and its possible interpretation is mentioned below. For each claim, clearly indicate whether you agree or disagree with the given interpretation

a.      “Sugar-free”: the food items with this label are less in calories and therefore useful for weight loss. agree/disagree/unsure

b.     “Cholesterol-free”: the food items with this label do not contain cholesterol. However, they can still increase blood cholesterol. agree/disagree/unsure

c.      “Low in glycemic load and low in glycemic index”: the food items with this label are safe to consume as much as one wants. agree/disagree/unsure  

d.     “Zero/no trans-fats”: the food item with this label can have trans fats in it, although in a very small amount. agree/disagree/unsure

6.     Which of the following nutrients increase bad cholesterol in the body? Choose the appropriate option for each given nutrient.

Sugar: yes/no/don’t know

Saturated fats: yes/no/don’t know

Omega-3 PUFA fats: yes/no/don’t know

Trans fats: yes/no/don’t know

Monounsaturated fats (MUFA): yes/no/don’t know

7.     At your college canteen, the available food options are not healthy. You depend on your college canteen for afternoon snacks. What would be your take on this in order to ensure that you don’t always eat unhealthy snacks? (Give at least two alternatives)

1)

2)

Table 7 gives glycemic load and glycemic index values of several food items. Refer to this table to answer questions 8, 9 and 10.

**Table 7:** Glycemic load and glycemic index of selected food items

Food item	Glycemic index (as % of glucose)	Glycemic load per regular serving
White bread	70	20
Potato, baked	85	26
Mango	56	8

8.     Which food item will release sugar in the blood at the fastest speed? …………………………………………………………………

9.     Which food item will have the largest effect on its sugar content in the blood? …………………………………………………………………

10.  Single serving of which food item is a better choice for a diabetic person if they have to choose from the given options? …………………………………………………………………

11.  Mention true/false:

“Switching to fruits and vegetables-based diet with protein supplements is the healthiest way to shed extra weight and/or to stay slim.”: true/false

“In order to get best quality protein one must take eggs and non-vegetarian food items.”: true/false

“Oils having a high amount of polyunsaturated fatty acids (PUFA) are not necessarily good for heart health.”: true/false

“Oils and fats are the main culprits that lead to overweight and obesity. Therefore, avoiding fried food items and butter/ghee is the best way to lose weight.”: true/false

12.  Write one disadvantage of Omega-6 polyunsaturated fatty acids (PUFA).

…………………………………………………………………

13.  The table below gives the nutritional information available on the product label of edible cooking oils in India. From the given information which oil do you think is a better choice from the health perspective? Put a circle on the option of your choice.

**Table 8:** Nutrition information given on food labels for edible oils

Name of the oil	Saturated fats (as % of total fat)	MUFA (as % of total fat)	PUFA (as % of total fat)	Transfats (as % of total fat)	Health claims/remarks
Groundnut oil	20	54	26	0	-
Sunflower oil	9	25	66	0	Less absorption of oil while cooking Rich in PUFA
Ricebran oil	24	40	34	2	Heart friendly cooking oil

14.  Give at least one benefit of physical activities and/or exercise in the following scenarios

**Table 9:** Write down benefits of exercise in given scenarios

Scenarios	Benefits
preventing/managing diabetes	
preventing/managing dyslipidemia and/or high blood cholesterol	

15.  Imagine that your aunt, aged 57 years, and overweight is diagnosed with borderline diabetes. She is advised to follow certain lifestyle changes including exercise. She tried walking for a few days, but she started getting knee joint pain and lost her motivation for exercise. What alternative strategy would you suggest she incorporate exercise into her routine?

16.  Write at least two disadvantages of eating refined flour such as maida.

       1) ………………………………………………………………

      2) ………………………………………………………………

17.  Imagine that your uncle, 63 years has been diagnosed with dyslipidemia (high total blood cholesterol and high LDL). His BMI is 28. He is taking appropriate medicines as per the physician’s advice. He is also trying to follow several dietary and lifestyle changes as per the physician’s advice. However, he finds it difficult to decide which food items are appropriate for him. Can you help him choose a healthier food item from the following options?

Set: 1

a.      Puri (mathri) made from whole wheat flour and groundnut oil.

b.     Sev-mamra made in palmolein oil.

Set: 2

a.      Skimmed milk.

b.     Whole milk.

c.      Milk with less than 3% fat.

Set: 3

a.      Cashew nuts.

b.     Walnuts.

18.  Mention at least three sources of Omega-3 PUFA (polyunsaturated fatty acids) in the diet.

1)……………………………….

2)……………………………….

3)……………………………….

19.  The fat present in milk and milk products is largely ……………………..

a.   Saturated fat                                       b.   cholesterol

c.   Polyunsaturated fat                             d.   monounsaturated fat

20.  The fat present in chicken and meat such as mutton is largely……………………………

a.   Saturated fat                                       b.   Omega 6

c.   Omega 3                                              d.   monounsaturated fatty acids (MUFA)

21.  Read the following passage and answer the questions given below the passage:

“After rice, I feel ghee occupies the unenviable position as one of the most misunderstood foods in India today. At one time considered the food of Gods, it’s now a “fattening” ingredient and somehow responsible for the lifestyle diseases of this generation. But is that the truth? Since the 70s and 80s when inspired by the marketing and propaganda of “heart healthy” vegetable oils, an entire country let go of its 5000-year old food wisdom to eat ghee, has our heart health really improved? Are there fewer cases now of diabetes, high cholesterol, etc.? Or did we make a blunder when Ghee was labeled “saturated fat” and pushed in the same category as trans fats and hydrogenated fats?

Here is the summary of “The goodness of ghee” series I ran on Facebook and Twitter last week:

“Ghee is fattening” - ghee by nature is lipolytic, that which breaks down fat. And this is due to its unique short-chain fatty acid structure. “Ghee is a saturated fat” - It's saturated fat, yes, but with such a unique structure that it actually helps mobilize fats from stubborn fat areas of the body. Not a saturated fat like trans-fats in your biscuits, cakes, pizza, etc.

Additionally, ghee has antibacterial and antiviral properties. Other than helping you recover from sickness, it ensures that you don't fall sick. And the antioxidants in ghee make it the miraculous anti-wrinkling and anti-aging therapy you were searching for.

What does our ancient food wisdom tells us: Runam krutva, ghrutam pibet - take a loan, but drink ghee. Cook in it or add on top of cooked food, it will continue to bless you.”

(Excerpted from http://rujutadiwekar.blogspot.com/2013/03/the-goodness-of-ghee.html )

Think critically about this advice. Do you agree with what is said? Why? Do you disagree with anything being said here? What is it that you disagree with? Why?

22.   Please clearly indicate whether you found this information interesting and useful. If yes, specify how. Also please feel free to give your suggestions and feedback on the content and its relevance to you.
